# Pathways of airway oxidant formation by house dust mite allergens and viral RNA converge through myosin motors, pannexons and Toll‐like receptor 4

**DOI:** 10.1002/iid3.216

**Published:** 2018-03-15

**Authors:** Jihui Zhang, Jie Chen, Shannon C. Mangat, Chathuri Perera Baruhupolage, David R. Garrod, Clive Robinson

**Affiliations:** ^1^ Institute for Infection and Immunity St George's, University of London London UK; ^2^ Faculty of Biology, Medicine and Health University of Manchester Manchester UK

**Keywords:** Airway epithelium, cysteine protease, house dust mite allergens, pannexons, reactive oxidant species, toll‐like receptor 4

## Abstract

**Introduction:**

Intracellular reactive oxidant species (ROS) are generated in human airway epithelial cells by the prothrombinase action of Group 1 house dust mite (HDM) allergens and by ligation of viral RNA sensor Toll‐like receptors (TLRs). We explored signaling convergence between HDM allergens and TLRs in ROS generation because epithelial cells form the primary barrier against inhaled substances and dictate host responses to allergens and viruses.

**Methods:**

ROS formation by Calu‐3 human airway cells was studied by measuring dihydrorhodamine 123 oxidation after activation by polyinosinic:polycytidylic acid (to activate TLR3), CL097 (to activate TLR7), a natural mixture of HDM allergens, or BzATP.

**Results:**

TLR4 activation was identified as an indispensable response element for all stimuli, operating downstream from myosin motor activation, pannexon gating for ATP release and the endogenous activation of prothrombin. Exogenous prothrombin activation by HDM allergens was prevented by SGUL 1733, a novel inhibitor of the proteolytic activity of Group 1 HDM allergens, which thus prevented TLR4 from being activated at source.

**Conclusions:**

Our data identify for the first time that endogenously‐generated prothrombin and TLR4 form a shared effector mechanism essential to intracellular ROS generation activated by a group 1 HDM allergen (itself a prothrombinase) or by ligation of viral RNA‐sensing TLRs. These stimuli operate a confluent signaling pathway in which myosin motors, gating of pannexons, and ADAM 10 lead to prothrombin‐dependent activation of TLR4 with a recycling activation of pannexons.

## Introduction

The archetype of Group 1 house dust mite (HDM) allergens, Der p 1, is an inhalant allergen of global clinical significance. It is a potent stimulus for the intracellular production of reactive oxidant species (ROS) in human airway epithelial cells and the most significant component in the constellation of HDM allergens with this bioactivity 1, 2, 3. This ROS generation which is inter alia of mitochondrial origin, is an innate response to the allergen and is triggered as a consequence of the recently discovered prothrombinase character of Der p 1 3. Group 1 HDM allergens are cysteine peptidases with highly conserved identities and functional activities across HDM species 1 so it is reasonable to infer from the behaviour of Der p 1, the group 1 allergen from *Dermatophagoides pteronyssinus*, that this prothrombinase activity is exhibited by all Group 1 HDM allergens. Consequently, all Group 1 HDM allergens are vulnerable to Allergen Delivery Inhibitor (ADI) drugs which have been designed as a novel inhaled approach asthma treatment 1, 2, 3. The generation of thrombin by Der p 1 liberates the canonical activator of protease activated receptor (PAR) 1 and PAR 4 and is necessary for ROS production 3. The full blockade of ROS production by antagonism of either receptor suggests that these PARs are activated as heterodimers or hetero‐oligomers 3, analogous to a behavior identified in platelets 4, 5.

Downstream from the activation of PAR 1 and PAR 4 by the Der p 1‐thrombin axis is the release of ATP through pannexon channels 3, 6. Additional elements of the signaling between Der p 1 and ROS generation have been defined and include a major role for a disintegrin and metalloprotease (ADAM) 10 6. The ability of Der p 1 to trigger ROS production innately through a thrombin‐dependent mechanism casts new light on how allergens directly influence the Th2 bias of immune responses and have the potential to drive the proliferation of smooth muscle 3, 6. ROS induce histone modifications and the activation of redox‐sensitive transcription factors which promote pro‐allergic cytokines. Furthermore, by direct protein modification they activate signal transduction cascades (e.g., mitogen‐activated protein kinases and the signal transducer and activation of transcription family) associated with allergy and asthma 7.

One notable aspect of epithelial ROS is their ability to regulate the release of interleukin 33 (IL‐33) which is enhanced in asthma 8, 9. Significantly, constitutive IL‐33 and the Th2 responses it directs inhibit innate defence against respiratory viruses 3, 8, 10. Intriguingly, we previously identified intracellular ROS production in epithelial cells which was dependent upon ATP release following the stimulation of the viral RNA sensors Toll‐like receptor (TLR) 3, melanoma differentiation associated protein‐5 (MDA‐5) and retinoic acid inducible gene‐I (RIG‐I) 3. Similarities between ROS generation initiated by virus detection and HDM allergens are interesting because interactions between asthma triggers and respiratory viruses are risk factors for asthma exacerbations. In this context it is significant that anti‐oxidant deficits are established risk factors for asthma and correlate with asthma severity 7, 11.

Studies in mice identify TLR4 expression in the airway epithelium as indispensable for allergic sensitisation and implicate it as an activator of IL‐1α production which triggers the autocrine release of granulocyte‐macrophage colony stimulating factor (GM‐CSF) and IL‐33 12, 13. As IL‐33 release is ROS‐regulated, in part, by NADPH oxidase dual oxidase 1 (DUOX1) whose activity is elevated in allergic disease 9, we were interested to explore the activation of TLR4 by signaling mechanisms operated by Der p 1 and ligation of viral RNA‐sensing TLRs. Specifically, we sought to define components leading to TLR4 activation and postulated that pannexons, and their associated mechanisms that gate ATP release, are key points where convergence between allergen and viral RNA stimuli might create a confluent pathway to TLR4 activation.

## Methods

### Chemicals and reagents

CL 097 (2‐(ethoxymethyl)‐1H‐imidazo(4,5‐c)quinolin‐4‐amine) was from InVivogen (Toulouse, France). TAK‐242, (ethyl‐(6*R*)‐6‐(N‐(2‐chloro‐4‐fluorophenyl)sulphamoyl)cyclohex‐1‐ene‐1‐carboxylate was from Merck Millipore (Watford, Hertfordshire, UK). ML7 (hexahydro‐1‐[(5‐iodo‐1‐naphthalenyl)sulphonyl]‐1*H*‐1,4‐diazepine hydrochloride); glycyl H1152 ((*S*)‐(+)‐4‐glycyl‐2‐methyl‐1‐[(4‐methyl‐5‐isoquinolinyl)sulphonyl]‐hexahydro‐1*H*‐1,4‐diazepine dihydrochloride; GI 254023X ((2*R*)‐*N*‐[(1*S*)‐2,2‐dimethyl‐1‐[(methylamino)carbonyl]propyl]‐2‐[(1*S*)‐1‐(*N*‐hydroxyformamido)ethyl]‐5‐phenylpentanamide); AG1478 (*N*‐(3‐chlorophenyl)‐6,7‐dimethoxy‐4‐quinazolinanine hydrochloride); CP 690550 ((3*R*,4*R*)‐4‐methyl‐3‐(methyl‐7*H*‐pyrrolo[2,3‐*d*]pyrimidin‐4‐ylamino)‐β‐oxo‐1‐piperidinepropanenitrile citrate, and AZ10606120 (*N*‐[2‐[[2‐[(2‐hydroxyethyl)amino]ethyl]amino]‐5‐quinolinyl]‐2‐tricyclo[3.3.1.13,7]dec‐1‐ylacetamide dihydrochloride were obtained from Tocris (Avonmouth, Bristol, UK). Argatroban ((2R,4R)‐1‐[(2S)‐5‐[(aminoiminomethyl)amino]‐1‐oxo‐2‐[[(1,2,3,4‐tetrahydro‐3‐methyl‐8‐quinolinyl)sulphonyl]amino]pentyl]‐4‐methyl‐2‐piperidinecarboxylic acid; formoterol fumarate dihydrate ((R*,R*)‐N‐[2‐hydroxy‐5‐[1‐hydroxy‐2‐[[2‐(4‐methoxyphenyl)‐1‐methylethyl]amino]ethyl]phenyl]formamide; salbutamol (α^1^‐[[(1,1‐dimethylethyl)amino]methyl]‐4‐hydroxy‐1,3‐benzenedimethanol hemisulphate); BzATP (2′(3′)‐O‐(4‐benzoylbenzoyl)adenosine 5′‐triphosphate triethylammonium salt); UTP (uridine‐5′‐triphosphate); cycloheximide and monensin were from Sigma–Aldrich, Poole, Dorset, UK. Dihydrorhodamine‐123 was obtained from Life Technologies (Paisley, Renfrewshire, UK). SGUL 1773 (N‐((S)‐1‐(((S)‐1‐(((S)‐1‐(benzylamino)‐4‐methyl‐1,2‐dioxopentan‐3‐yl)amino)‐oxopropan‐2‐yl)amino)‐3,3‐dimethyl‐1‐oxobutan‐2‐yl) benzamide), a potent (K_i_ <5 nM) and selective inhibitor of Der p 1 and other Group 1 HDM allergens was synthesized as described in our previous work 2. This compound has no significant inhibitory activity against thrombin (IC_50_ > 100 μM).

Polyinosinic:polycytidylic acid, 1.5–8 kb (InvivoGen), was used routinely, with comparative studies being made with a shorter form of poly i:c (0.2–1 kb) where stated. Antibodies for use in fluorescent antibody labeling or western blotting were from AbD Serotec (Kidlington, Oxfordshire, UK), Abcam (Cambridge, UK), and Insight Biotechnology (London, UK). An Alexa 488‐conjugated secondary antibody for immunofluorescence detection was from Life Technologies. Consumables for SDS–PAGE and immunoblotting were from InVitrogen/ThermoFisher (Paisley, Renfrewshire, UK) and GE Healthcare (Little Chalfont, Buckinghamshire, UK). Fluorescent antibody labeling and immunoblotting were performed conventionally. TLR3 and TLR4 were immunoblotted using polyclonal antibodies directed against, respectively, a 15 amino acid synthetic peptide from the carboxy terminus and an epitope corresponding to residues 242–321 in the human receptors. TLR7 was detected by blotting using a monoclonal antibody raised against recombinantly expressed residues 451–500 from the human receptor sequence. Immunoblotting of pannexin‐1 was undertaken using a polyclonal antibody directed against a conjugate of residues found within the span 150–250 in the human protein. Detection of EGFR employed a monoclonal antibody raised against a recombinant protein corresponding to amino acids 363–499 in the human sequence. MYLK was immunoblotted using a monoclonal antibody directed to a human peptide sequence and which is known to detect non‐muscle MYLK isoforms. Immunoblotting of prothrombin was undertaken with a polyclonal antibody raised against full length protein from human plasma. ADAM 10 was detected by monoclonal antibody raised against residues 1–300 of the human enzyme, while ADAM 17 was immunoblotted using a polyclonal antibody directed against a synthetic peptide from the C‐terminus sequence. All immunoblotting was performed with sample loadings normalized for protein concentration.

Cell culture media and reagents were obtained from Life Technologies, Sigma–Aldrich, and GE Healthcare. Transfection reagents and siRNA duplexes for gene silencing studies (usually these were mixtures of 3 target‐specific 19–25 nt siRNAs or scrambled controls against no known targets) were provided by Insight Biotechnology.

### HDM allergen harvesting and Der p 1 purification

*D. pteronyssinus* HDM were maintained in continuous solid‐phase culture under barrier conditions with controlled temperature (25°C) and relative humidity (75%) in custom‐engineered containment. Mixed, native HDM allergens in their natural proportions were prepared according to our routine processes. ELISA measurements (Indoor Biotechnologies, Cardiff, UK) were used to determine the Der p 1 content of the allergen extracts, whilst the catalytic activity of Der p 1 was quantified as described elsewhere using a fluorescence resonance energy transfer substrate designed for Der p 1 2, 14. We elected to use mixed HDM allergens for the majority of these studies because this presentation is most representative of the material to which the airway epithelium is exposed in life. HDM mixtures were normalized by reference to Der p 1 content expressed as μg/mL^−1^ as previously described 3. Thus ‘HDM 1’ is equivalent to 1 μg/mL^−1^ Der p 1 and so forth. For consistent batch to batch proteolytic activity of Der p 1 in allergen preparations, experiments were conducted in the presence of 5 mM L‐cysteine which was present also in vehicle controls in these experiments.

To provide a source of reference Der p 1 and to generate protein for confirmatory studies, the mixed HDM allergens were used as feedstock for protein purification. Der p 1 was purified from harvested mite culture medium at 4°C by adding 2–3 volumes of Dulbecco's PBS followed by overnight stirring. The supernatant was collected after centrifugation (30 min, 24,000*g*, 4°C) and solid ammonium sulphate added to 50% saturation in the presence of 1 mM EDTA. Pellets were collected by centrifugation after a minimum of 2 h precipitation. Insoluble matter was removed from the reconstituted solution and the soluble fraction then subjected to size exclusion chromatography on an ÄktaPurifier system (HiPrep 16/60 Sephacryl S‐200 HR, GE Healthcare) in elution buffer (0.2 M sodium phosphate containing 0.5 M sodium chloride and 1 mM EDTA, pH 7.4). A fraction with retention volume 90–120 mL was collected and eluted through a soybean trypsin inhibitor (SBTI) column. After concentrating the eluate it was re‐chromatographed on Sephacryl S‐200 with a further round of SBTI affinity chromatography. The eluate from this was then concentrated and desalted using an Amicon ultrafiltration cell (Millipore, Bedford, MA) equipped with 10 kDa cut‐off membrane. The desalted concentrate was diluted into 20 mM Tris‐HCl buffer, pH 8.0, for ÄktaPurifier chromatography on Resource Q (GE Healthcare) from which Der p 1 was eluted by a 0–0.5 M sodium chloride gradient. Peaks containing Der p 1 were analyzed by SDS–PAGE and MALDI‐TOF mass spectrometry (Bruker Flex, Bruker, Coventry, UK) and combined. The quantity of Der p 1 was determined in a quartz cuvette by absorbance at 280 nm using an extinction coefficient of 47,705 M^−1^ cm^−1^.

### Cell culture and transfection

Calu‐3 cells were cultured according to standard methods previously disclosed 15, 16, 17. This cell line preserves the integrity of tight junctions (TJs) and readily forms polarized monolayers which develop substantial transepithelial resistance 15, 16. Our extensive prior work has established that this cell line responds to HDM allergens through a variety of mechanisms also shown to operate in primary cultures of human airway epithelial cells 17, 18, 19, 20. Notably, intracellular ROS generation which is dependent upon the cleavage of PAR1 and PAR4 by Der p 1 is a feature we have already identified in primary cultures from human lung and in other immortalized epithelial cell lines from human airway 3. Gene silencing experiments with siRNA duplexes were performed according to the supplier protocol optimized for these experiments. In some experiments it was observed that the transfection process per se had small effects on the maximal rate of oxidant of stimulated ROS production. For the purposes of interpretation, the effects of active interventions have been considered with reference to the corresponding transfection controls and not the “clean” non‐transfected controls.

### Measurement of ROS production

ROS production was studied in cells plated into 96‐well format on clear‐bottomed black culture plates (Corning, Amsterdam, The Netherlands). Cells were washed and then loaded for 15 min at ambient temperature with dihydrorhodamine–123 (10 μM) in phosphate buffered saline (PBS), after which excess probe was removed by washing and the PBS replaced by Hanks’ balanced salt solution (HBSS) containing 20 mM 4‐(2‐hydroxyethyl)‐1‐piperazine ethanesulphonic acid (HEPES). Where appropriate, cells were then treated with inhibitors for 20 min at 37°C prior to the addition of stimulating agents (HDM allergen mixture, purified Der p 1, poly i:c, CL 097, BzATP, or UTP). Drug vehicles comprised HBSS/HEPES containing, where appropriate, any aprotic solvents used to stored concentrated solutions of drug substances. Dilution schemes ensured that only trace amounts of these solvents entered experiments. Controls were also incorporated to examine compounds for baseline effects on ROS generation.

Reactions were started by the addition of the stimulant and maintained at 30°C under constant humidity in an Envision plate reader (Perkin Elmer, Seer Green, Buckinghamshire, UK) for the duration of the experiment. Fluorescence measurements were made every 5 min (excitation 485 nm, emission 535 nm) and the maximum rate of oxidant production determined from the progress curves (increase in fluorescence upon oxidation of dihydrorhodamine‐123 to rhodamine) recorded for each well over a 2.5 h period. The oxidative formation of rhodamine may be accomplished by a range of reactive species generated by different mechanisms, so without prejudice we electively refer to these measurements as intracellular ROS to reflect contributions from multiple sources. Advantageously, dihydrorhodamine accumulates preferentially in mitochondria and our previous work using a DNA‐binding triphenylphosphonium analogue of dihydroethidium and a mitochondrial disruptor indicates that a component of this ROS generation is of mitochondrial origin via formation of $O_{2}^{-}$ 3. Additionally, dihydrorhodamine is an effective reporter of products formed by the facile, spontaneous decomposition of peroxynitrite arising through the reaction of $O_{2}^{-}$ with nitric oxide. Nitric oxide is formed in airway epithelial cells as a consequence of ATP‐dependent activation of P_2_X_7_ and P_2_Y_2_ receptors (our unpublished observations). This observation does not preclude the tandem formation of ROS derived from NOX/DUOX activity in the response to allergen or viral RNA sensor stimulation.

Standard concentrations of stimulants used for cell activation were determined from previous experiments or concentration‐response relationships shown herein 3, 6 and unpublished data). Experiments involving stimulation by HDM allergen mixtures were routinely run using “HDM 1” for cell activation, but ROS generation is significant from concentrations at least 100‐fold lower. The relevance of this concentration range is based upon modeling the deposition and dissolution characteristics of HDM fecal pellets (which contain the allergenic proteins) in the surface liquid of human airways. Given the dimensions of the bronchial tree of adults, and that from aerodynamic particle limitations HDM fecal pellets are unlikely to penetrate beyond third generation bronchi, the epithelial area exposed in real life would be ∼143 cm^2^. Taking account of the rate of HDM fecal pellet inhalation, the growth surface of a well in a culture plate (96 well format) and corrections for the differences in liquid height between culture conditions and airway surface liquid, this suggests a well would require a *theoretical average* of 400 HDM fecal pellet equivalents to mimic natural exposure. However, the escalating rate of inertial impaction in lower‐generation airways will create regional inhomogeneities where the actual allergen burden is expected to be significantly in excess of the theoretical. From ELISA determinations, taking an average mass amount of Der p 1 in a single fecal pellet to be 0.1 ng, the theoretical average exposure equates to 40 ng Der p 1 under the stated conditions. This lies within the observed range of ROS generating ability for calu‐3 cells or primary cultures which spans the range 2–200 ng Der p 1 per well 3, 6.

### Statistical analyses

Unless stated otherwise, data are presented as mean ± s.e.m (*n* = 8) in single experiments with cells from the same passage. These experiments were repeated >3 times with cells from different passages for confirmation and data were accepted only if repetition yielded similar outcomes, that is, the inferences that could be drawn were the same. Graphical representations have been made using bar and line charts for visual simplicity and because analysis of scatter did not reveal unusual or interesting aspects of the data not obvious from this form of display. Significance was determined using one‐way analysis of variance with Newman–Keuls post hoc testing in SigmaPlot v12. The threshold for statistical significance was *P* < 0.05 but in the majority of cases group differences exceeded this threshold and were highly significant, that is, *P *< 0.001. For convenience, the labeling scheme used in the Figures indicates *P *< 0.05 even when analyses indicated higher levels of significance.

### Ethical approval

No humans or animals were used in these experiments, so formal ethical approvals were not required.

## Results

### TLR3‐dependent ROS generation in airway epithelial cells requires ATP and pannexin‐1‐containing pannexons

**Figure 1:**
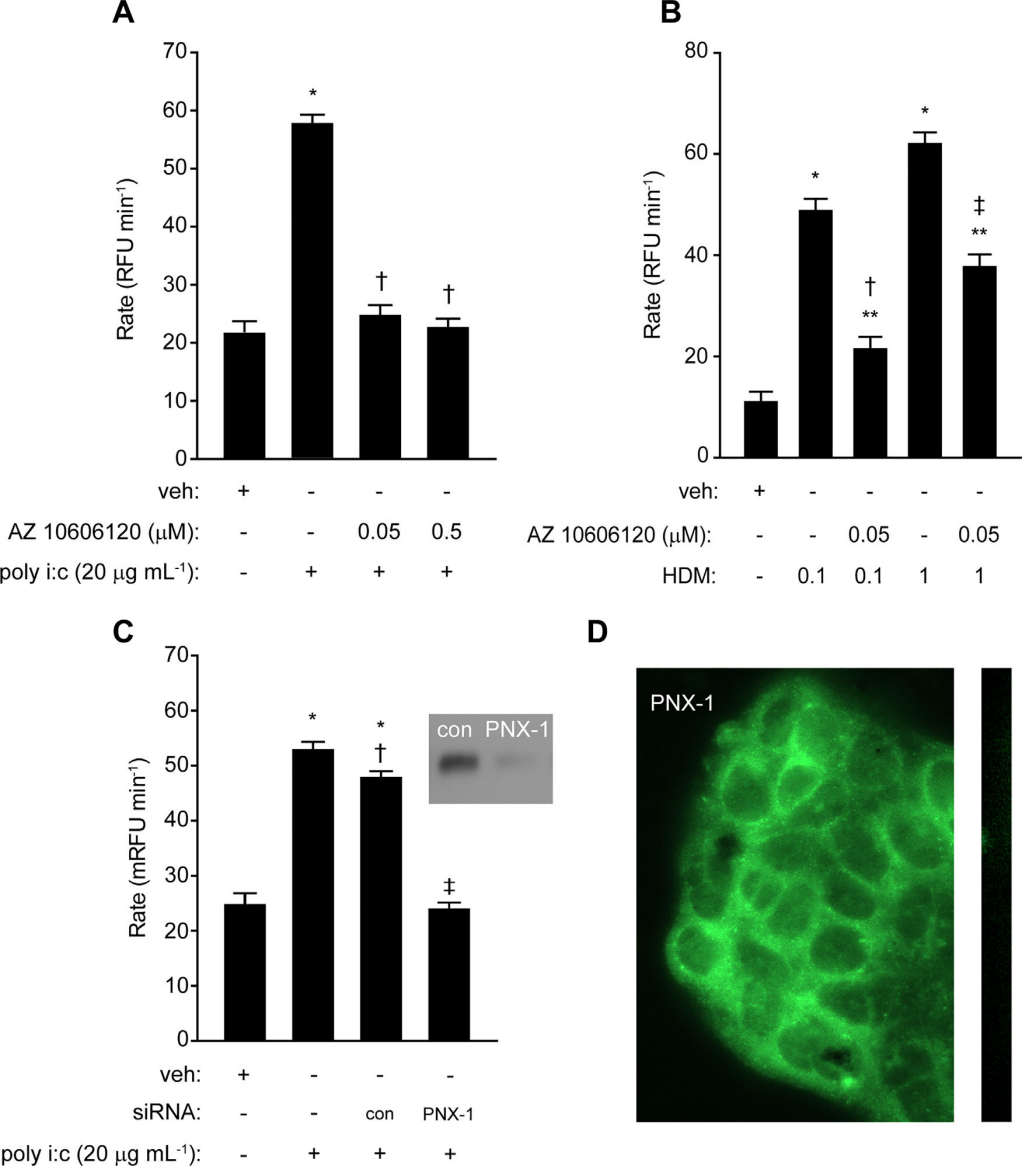
Intracellular ROS production by poly i:c depends on ATP release and pannexons. (A) Inhibition of poly i:c induced ROS generation by AZ 10606120 (*P < 0.05 *v* vehicle control (veh), **P < 0.05 *v* poly i:c). (B) Inhibition by AZ 10606120 of ROS production initiated by mixed HDM allergens (*P < 0.05 *v* veh; **P < 0.05 *v* veh; ^†,‡^ *P* < 0.05 *v* corresponding HDM response). (C) Silencing of pannexin 1 (PNX‐1) by siRNA inhibits ROS production by poly i:c (*P < 0.05 *v* veh, ^†^ *P* < 0.05 *v* poly i:c without control transfection (con), ^‡^ *P* < 0.05 *v* poly i:c with or without control transfection). Inset image shows immunoblot of PNX‐1 in cells transfected with target siRNA or control siRNA (con). (D) Fluorescent antibody labeling of pannexin‐1 in calu‐3 cells. The strip image depicts a negative staining control.

Our initial approach was to investigate whether surrogates of viral infection behaved like Der p 1 in evoking intracellular ROS generation which was dependent upon pannexon‐gated ATP release 3. Exposure of cells to poly i:c, a ligand for TLR3 which acts as an endosomal sensor for double‐stranded viral RNA, resulted in a progressive and sustained generation of ROS. We first investigated the ability of poly i:c to generate ROS in an ATP‐dependent manner. To achieve this, cells were activated in the absence or presence of the negative allosteric modulator of P_2_X_7_ receptors, AZ 10606120, which provided a potent blockade of the response (Fig. 1A). For comparison, the effect of AZ 10606120 on responses to mixed HDM allergens is shown in Figure 1B. At present, the reasons for the incomplete inhibition of the response compared to cells stimulated with poly i:c are unclear, but this may be a function of an enduring cellular stimulation by the Group 1 protease allergen component of the allergen mixture. Our forerunner studies established that carbenoxolone, a well‐established inhibitor of signaling at connexons and pannexons, also attenuated the response to mixed HDM allergens or poly i:c (data not shown), consistent with ATP release/intercellular transfer occurring through a channel‐dependent mechanism. The lack of selectivity of carbenoxolone notwithstanding, a reasonable inference is that the channels responsible for the AZ 10606120‐inhibitable response are pannexons rather than connexons, although additional experiments are needed to confirm this. Nevertheless, this interpretation is supported by the inhibition of poly i:c response in cells in which pannexin‐1 expression was silenced by siRNA pre‐treatment (Fig. 1C) and recalls our previous work in which pannexin‐1 expression was shown to be crucial to the generation of intracellular ROS in airway epithelial cells stimulated by mixed HDM allergens 3. Fluorescent antibody labeling confirmed the presence of pannexin‐1 in calu‐3 cells which was expressed in a reticular pattern with a significant cytoplasmic background absent in labeling controls (Fig. 1D). Further work will be necessary formally to investigate whether connexon—mediated intracellular signaling contributes to the propagation of these responses, but given the diversity of connexin proteins this was considered beyond the scope of the present investigation.

### CL097‐dependent activation of intracellular ROS generation

**Figure 2:**
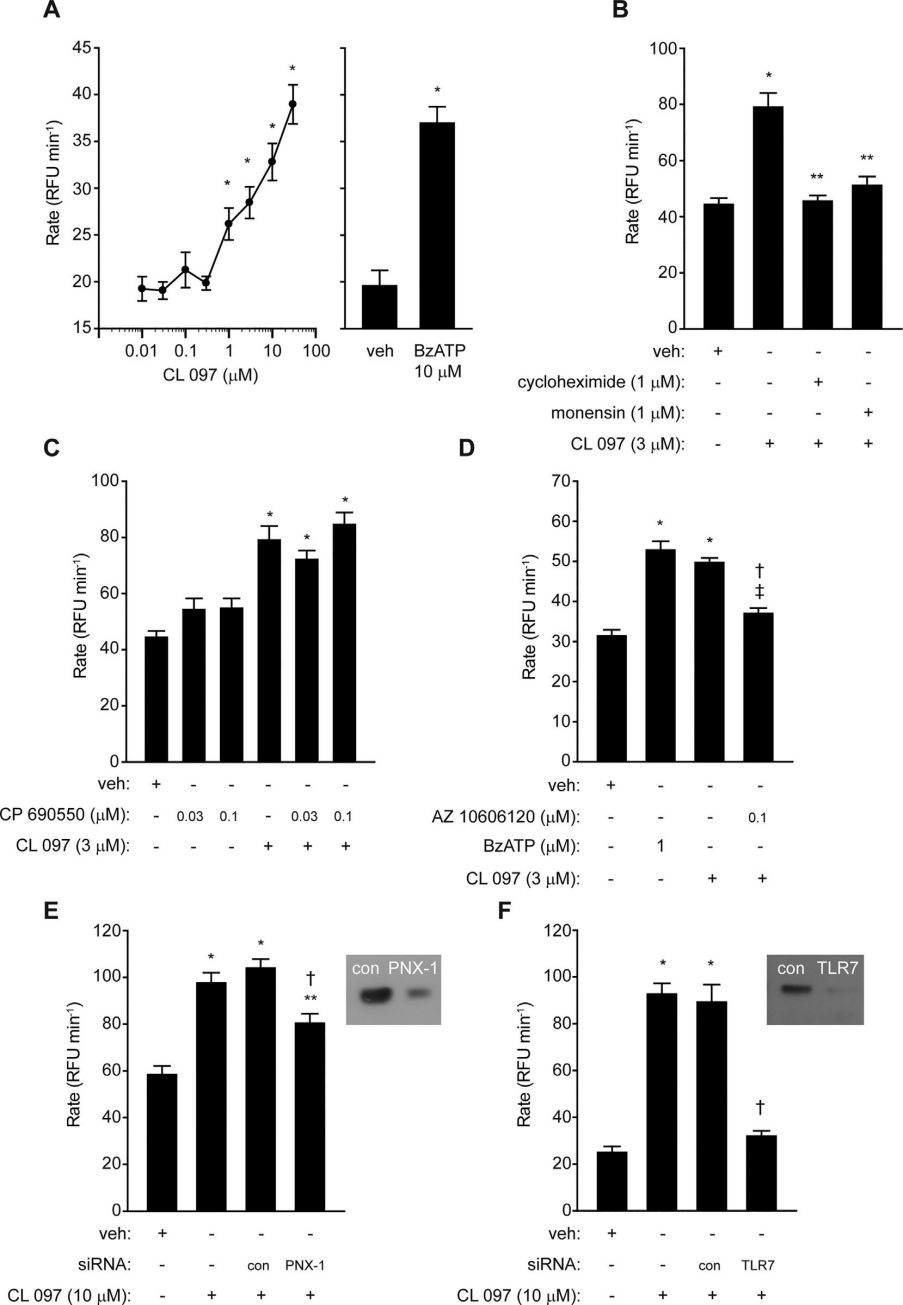
ROS production is stimulated by CL 097. (A) Concentration‐dependent effect of CL 097 referenced to the effect of BzATP (*P < 0.05 *v* control (veh)). (B) Inhibition of CL 097 by cycloheximide and monensin pre‐treatment (*P < 0.05 *v* veh, **P < 0.05 *v* CL 097). (C) Lack of effect of CP 690550 on CL 097 response (*P < 0.05 *v* veh). Inhibition of CL 097 by, D, AZ 10606120 (*P < 0.05 *v* veh, ^†^ *P* < 0.05 *v* CL 097, ^‡^ *P* < 0.05 *v* veh), E, gene silencing of pannexin‐1 (PNX‐1) (*P < 0.05 *v* veh, ^†^ *P* < 0.05 *v* CL 097 with or without transfection control (con), **P < 0.05 *v* veh), or F, TLR7 (*P < 0.05 *v* veh, ^†^ *P* < 0.05 *v* CL 097 with or without transfection control). Inset images in E,F show immunoblots of pannexin‐1 or TLR7 in cells transfected with the target siRNA (PNX‐1, TLR7) or control siRNA (con).

Having established that TLR3 ligation evoked ROS generation which was pannexon‐dependent, we then examined whether ligation of a sensor for single‐stranded RNA would evoke a response. CL097, a ligand for TLRs 7 and 8 which detect single‐stranded viral RNA, elicited an increase in ROS production which was concentration‐dependent and comparable to that evoked by BzATP which was used as a positive control (Fig. 2A). Inhibition by cycloheximide and monensin indicated that the action of CL097 was dependent upon protein synthesis and export (Fig. 2B), but the lack of effect of CP 690550, which was consistent with previous findings for poly i:c/TLR3 activation 3, suggested an independence from JAK signaling (Fig. 2C). However, the response was inhibited by AZ10606120 (Fig. 2D) and by transient silencing of pannexin‐1 expression (Fig. 2E). Gene silencing of TLR7 caused a marked decrease in ROS generation by CL097 (Fig. 2F).

### MYLK involvement in pannexon‐dependent ROS generation

**Figure 3:**
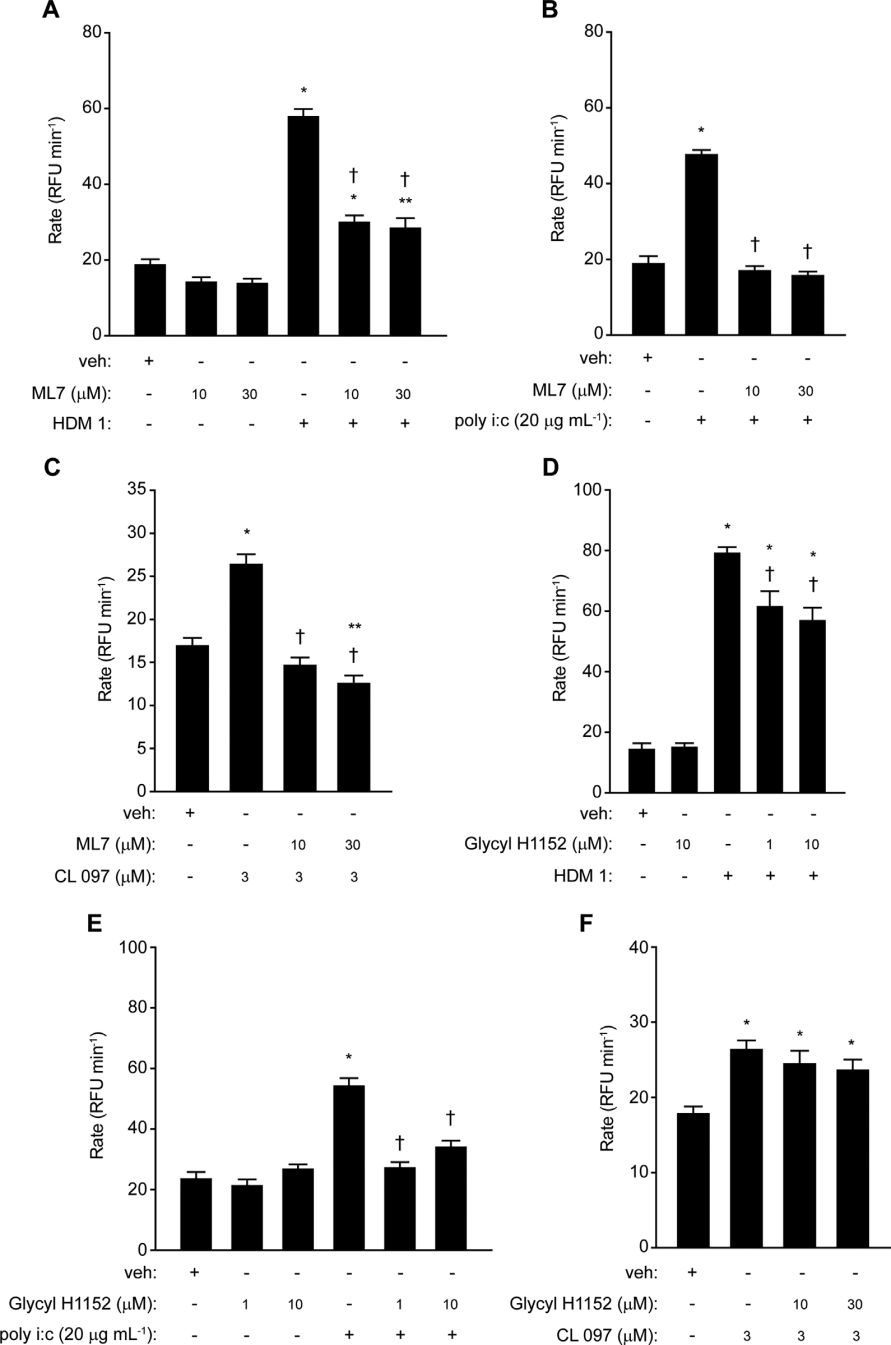
ROS production by HDM allergen mixture or poly i:c is dependent on myosin light chain kinase activity. Inhibition by ML7 of ROS generation by, A, mixed HDM allergens (*P < 0.05 *v* veh, **P < 0.05 *v* veh, ^†^ *P* < 0.05 *v* HDM 1), (B) poly i:c (*P < 0.05 *v* veh, ^†^ *P* < 0.05 *v* poly i:c), (C) CL 097 (*P < 0.05 *v* veh, ^†^ *P* < 0.05 *v* CL 097, **P < 0.05 *v* veh). Effects of glycyl H1152 on responses to, D, mixed HDM allergens (*P < 0.05 *v* veh, ^†^ *P* < 0.05 *v* HDM 1), (E) poly i:c (*P < 0.05 *v* veh, ^†^ *P* < 0.05 *v* poly i:c), and (F) CL 097 (*P < 0.05 *v* veh).

**Figure 4:**
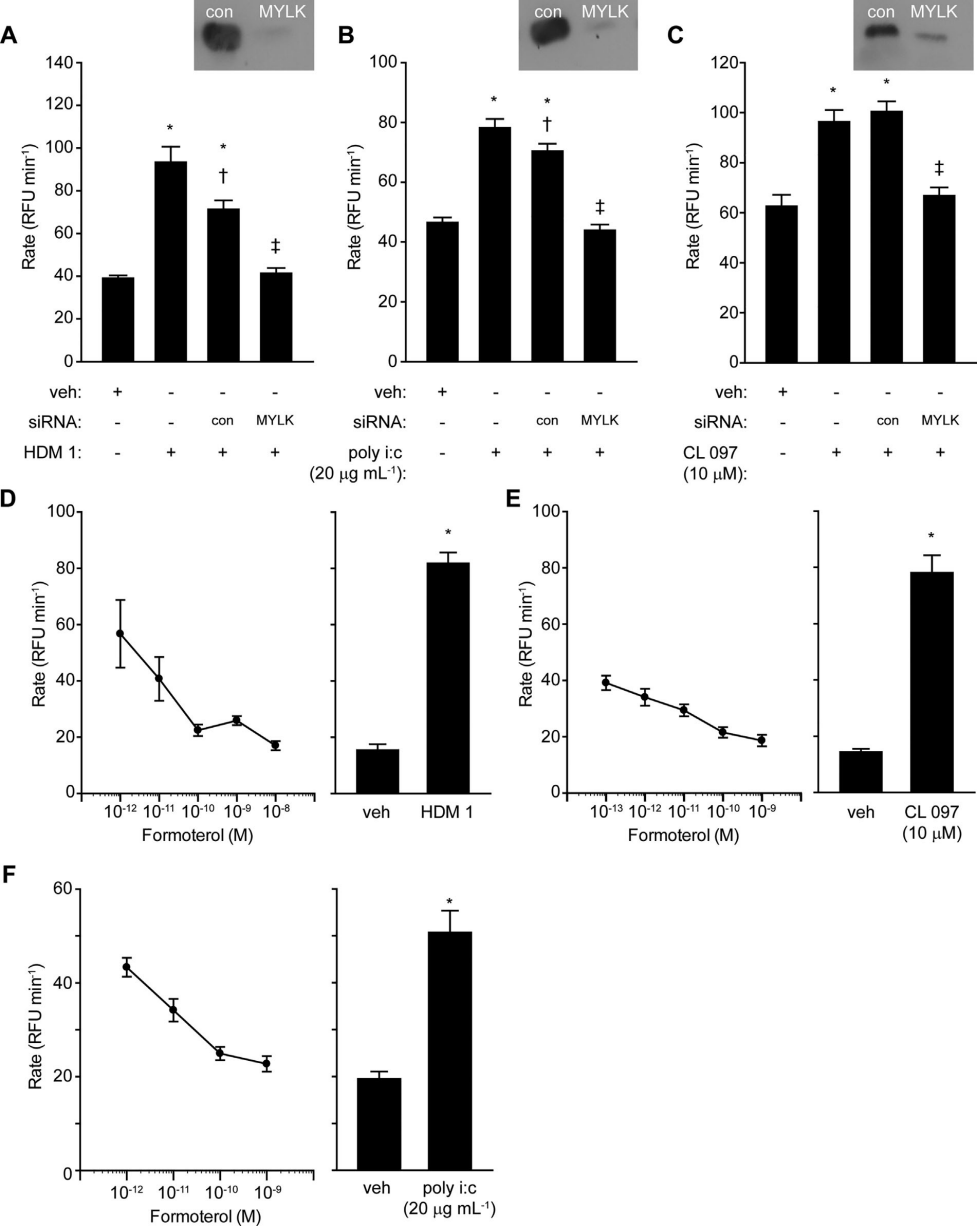
Modulation of MYLK activity inhibits intracellular ROS production in airway epithelial cells. (A–C) Knockdown of MYLK blocks ROS generation by HDM allergens, poly i:c or CL 097. For each stimulant *P < 0.05 *v* appropriate vehicle (veh), ^†^ *P* < 0.05 *v* corresponding stimulant in non‐transfected cells, ^‡^ *P* < 0.05 *v* appropriate stimulant with or without control (con) transfection. (D–F) Formoterol inhibits responses to HDM allergens, CL 097 or poly i:c. *P < 0.05 *v* veh for each agonist control. Inhibition by formoterol was significant (*P* < 0.05) over the range 10^−12^–10^−8 ^M with HDM allergen mixture or poly i:c activation and between 10^−13^ and 10^−9 ^M with CL 097.

With evidence that pannexon‐based signaling is an essential feature of ROS generation by the ligation of viral RNA sensors, we next investigated whether these channels were being operated in a manner similar to that initiated by HDM allergen stimulation. The gating of pannexons in non‐excitable tissues is not well understood, but may involve mechanical stress. Therefore, we were interested to establish whether ROS production activated by Der p 1, poly i:c or CL 097 involved myosin molecular motors and actin filaments. To do this we reasoned that interventions designed to prevent myosin light chain phosphorylation, either by inhibiting the action of myosin light chain kinase (MYLK) or Rho‐associated coiled‐coil kinases (ROCKs) which control the activity of myosin light chain phosphatase, should be informative. The MYLK inhibitor ML7 was without effect on baseline ROS production but blocked responses in cells stimulated by HDM allergens, poly i:c or CL 097 (Fig. 3A–C). The ROCK inhibitor glycyl H1152 had no effect on baseline ROS production but it attenuated responses to mixed HDM allergens or poly i:c (Fig. 3D,E). However, it was ineffective against CL 097 (Fig. 3F). Transient silencing with siRNA provided direct confirmation that MYLK‐regulated pathways are central to intracellular ROS generation activated by HDM allergen stimulation or the ligation of TLR3 or TLR7 (Fig. 4A–C).

As a further evaluation of the involvement of MYLK we treated cells with β_2_‐adrenoceptor agonists which are known, amongst other actions which may have a bearing on ROS production, to indirectly inhibit MYLK through its protein kinase A‐dependent phosphorylation. Formoterol produced a concentration‐dependent inhibition of all 3 stimuli (Fig. 4D–F), as did salbutamol albeit with lower potency (not shown).

### Viral RNA‐sensing TLRs utilize ADAM 10 and EGFR in ROS generation

The finding that the critical path to ROS production for all stimuli requires MYLK activation and actomyosin suggests that this step lies downstream from a point of signaling convergence which creates a confluent signaling mechanism. If correct, then other signaling events which appear downstream of MYLK and pannexon gating should exhibit similar conformity. Our previous work was the first to establish a role for ADAM 10 in the activation of intracellular ROS production by Group 1 HDM allergens 6, so we investigated whether ADAM 10 had a similar involvement in ROS generation in cells stimulated by ligation of viral RNA‐sensing receptors.

**Figure 5:**
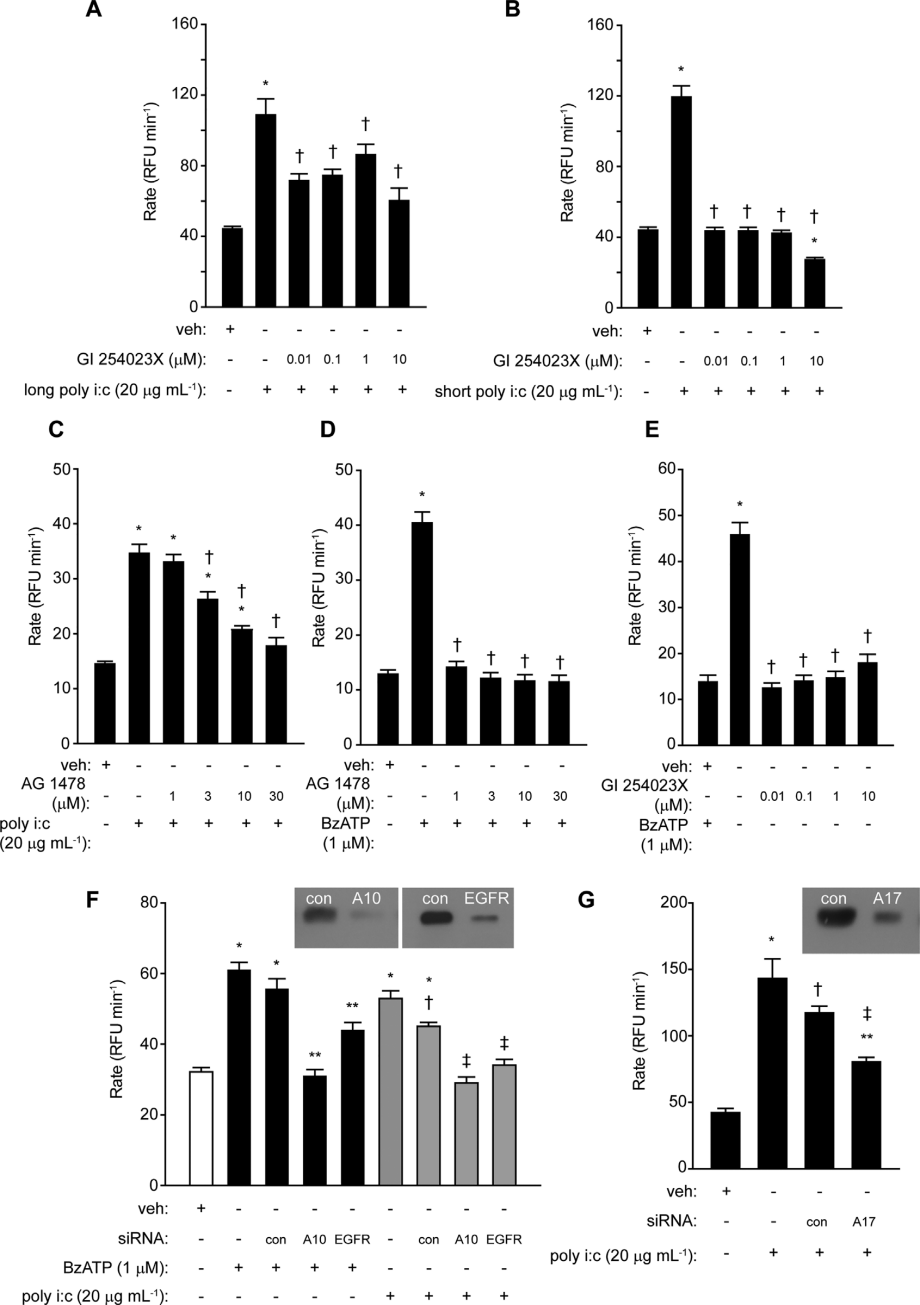
ROS generation by poly i:c requires ADAM 10 and EGFR activation. (A) Inhibition of responses to long poly i:c by GI 254023X (*P < 0.05 *v* vehicle (veh), ^†^ *P* < 0.05 *v* poly i:c). (B) Inhibition of responses to short poly i:c by GI 254023X (*P < 0.05 *v* veh, ^†^ *P* < 0.05 *v* poly i:c). (C) AG 1478 inhibits responses to poly i:c (*P < 0.05 *v* veh, ^†^ *P* < 0.05 *v* poly i:c). (D) AG 1478 inhibits responses to BzATP (*P < 0.05 *v* veh, ^†^P < 0.05 *v* BzATP). (E) Inhibition of responses to BzATP by GI 254023X (*P < 0.05 *v* veh, ^†^ *P* < 0.05 *v* BzATP). (F) Silencing of ADAM 10 (A10) or EGFR by siRNA blocks ROS generation by BzATP and poly i:c. For BzATP: *P < 0.05 *v* veh, **P < 0.05 *v* BzATP in non‐transfected or transfection control (con) cells. For poly i:c: *P < 0.05 *v* veh, ^†^P < 0.05 *v* poly i:c in non‐transfected cells, ^‡^ *P* < 0.05 *v* poly i:c with or without control transfection. (G) Response to poly i:c is inhibited by silencing of ADAM 17 (*P < 0.05 v veh, ^†^ *P* < 0.05 *v* poly i:c without transfection, ^‡^ *P* < 0.05 *v* poly i:c with and without control transfection, respectively and *P < 0.05 v veh). Inset images in F and G show immunoblots of ADAM 10, EGFR or ADAM 17 in cells transfected with the relevant target siRNA (A10, EGFR, A17) or control siRNA (con).

**Figure 6:**
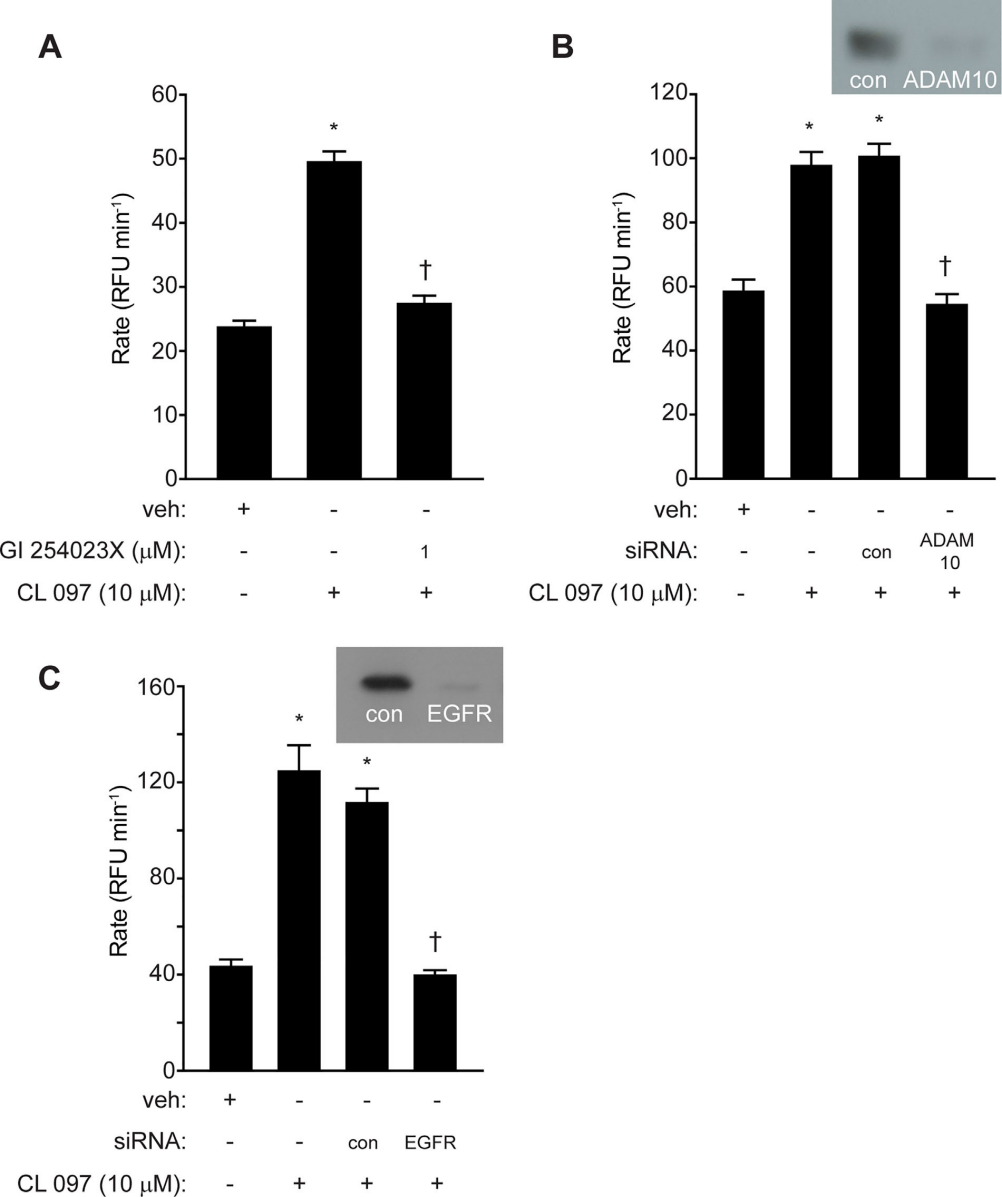
ROS generation by CL 097 requires ADAM 10 and EGFR activation. (A) Inhibition of CL 097 by GI 254023X (*P < 0.05 *v* veh, ^†^ *P* < 0.05 *v* CL 097). (B and C) Attenuation of responses to CL 097 by silencing of ADAM 10 or EGFR (*P < 0.05 *v* veh, ^†^ *P* < 0.05 *v* CL 097 with or without control (con) transfection. Inset images in B and C show immunoblots of ADAM 10 or EGFR in cells transfected with target siRNA or control siRNA (con).

The selective inhibitor of ADAM 10, GI 254023X, inhibited responses to both long and short forms of poly i:c, with a greater efficacy against the latter which our previous work 3 shows to be a more potent stimulant of responses mediated via RIG‐I (Fig. 5A,B). Activation of ROS production by poly i:c was inhibited by the EGFR tyrosine kinase inhibitor, AG 1478 (Fig. 5C), as were responses to BzATP (Fig. 5D). In addition, responses to BzATP were also blocked by GI 254023X (Fig. 5E). This suggests that, as with responses to HDM allergen stimulation reported elsewhere 6, the activation of ADAM 10 and EGFR lie downstream of ATP release and purinoceptor activation. ROS generation was prevented from rising above baseline levels following siRNA knockdown of either target (Fig. 5F), confirming the importance of both ADAM 10 and EGFR in signaling responses evoked by poly i:c. Knockdown of ADAM 17 was also capable of attenuating ROS production by poly i:c (Fig. 5G). Responses to CL 097 were similarly inhibited by GI 254023X and by siRNA directed against ADAM 10 or EGFR, consistent with intracellular ROS production triggered by the activation of TLR7 sharing downstream effectors with TLR3 responses (Fig. 6A–C).

### TLR3 and TLR7‐dependent ROS generation in airway epithelial cells requires prothrombin and thrombin

**Figure 7:**
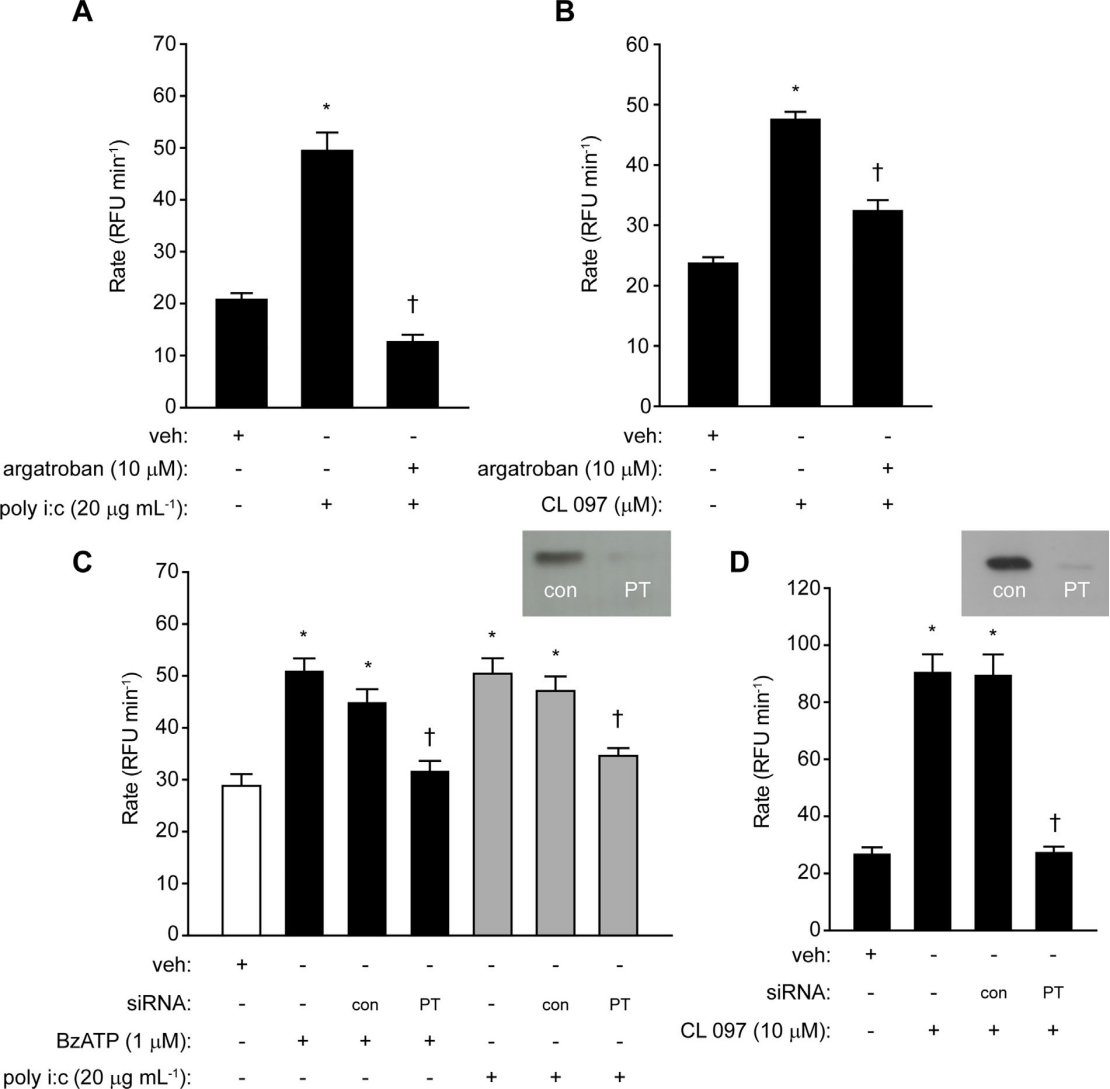
Intracellular ROS formation by poly i:c or CL 097 requires thrombin formation. (A and B) Inhibition by argatroban (*P < 0.05 *v* vehicle (veh), ^†^ *P* < 0.05 *v* poly i:c or CL 097). (C and D) Knockdown of prothrombin (PT) expression by siRNA blocks ROS generation in response to BzATP, poly i:c and CL 097 (*P < 0.05 *v* veh, ^†^ *P* < 0.05 *v* agonists in non‐transfected or transfection control (con) cells).

Treatment of cells with the thrombin inhibitor argatroban strongly attenuated ROS generation by poly i:c or CL 097 (Fig. 7A,B), suggesting that stimulation of TLR3 or TLR7 evokes the endogenous generation of thrombin. This was confirmed by siRNA silencing of prothrombin which effectively prevented any response to BzATP, poly i:c or CL 097 (Fig. 7C,D). While the recently‐discovered prothrombinase activity of Der p 1 establishes the rationale for thrombin formation through a direct, exogenously‐triggered innate response to HDM allergen exposure 3, this effect of BzATP, poly i:c or CL 097 was unanticipated and implies that downstream from pannexon opening and ATP release there is an endogenous trigger for the activation of prothrombin. Notwithstanding the uncertain identity of this trigger, this led us next to consider the possibility of a mechanistic linkage between these events and other innate receptor systems.

### TLR4 is activated by responses to Der p 1 and by ligation of TLR3 or TLR7

**Figure 8:**
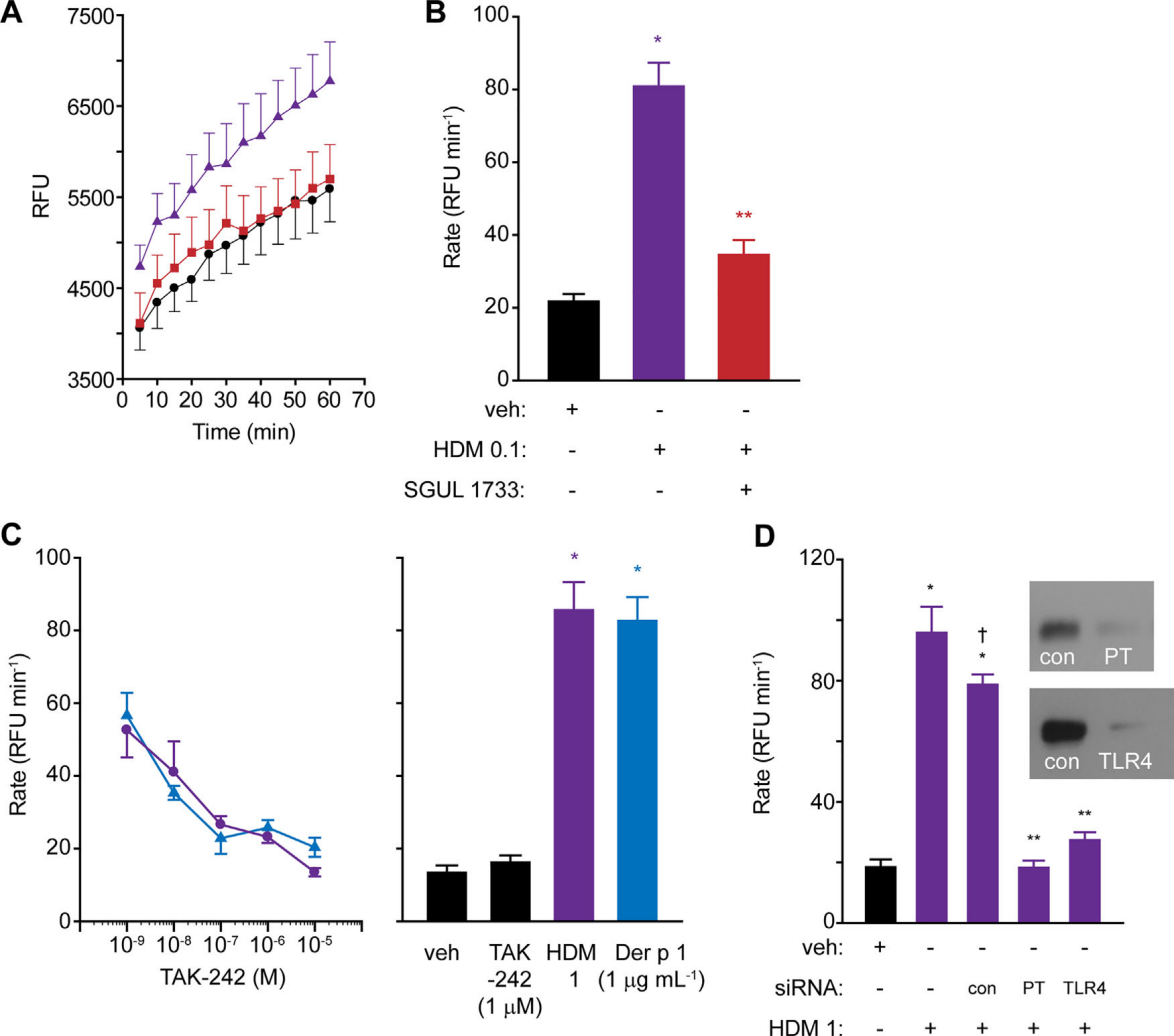
(A and B) Generation of ROS in calu‐3 cells by HDM allergens and its inhibition by allergen delivery inhibitor SGUL 1733. Panel A shows progress curves for ROS production in vehicle‐treated cells (black circles), cells treated with HDM 0.1 (purple triangles) and HDM‐treated cells in the presence of SGUL 1733 (red squares). Panel B shows the corresponding initial rates of ROS formation (*P < 0.05 *v* veh, **P < 0.05 *v* HDM 0.1). Data from quadruplicate observations thrice replicated. (C) Inhibition of responses to mixed HDM allergens (purple circles) or purified Der p 1 (blue triangles) by TAK‐242. All drug effects are *P* < 0.05 *v* the relevant positive control. Baseline ROS production in the absence or presence of TAK‐242 are shown in the bar chart, together with the positive control responses for the stimuli (*P < 0.05 *v* negative controls). (D) Cell silencing of prothrombin (PT) or TLR4 inhibits the response to mixed HDM allergens (*P < 0.05 *v* vehicle (veh), **P < 0.05 *v* HDM 1 with or without control (con) transfection, ^†^ *P* < 0.05 *v* HDM 1 in non‐transfected cells).

Activation of ROS production in airway epithelial cells by mixed HDM allergens from *D. pteronyssinus* was extensively inhibited by SGUL 1733, a potent and selective ADI compound which inhibits Der p 1 (Fig. 8A,B). Given the prothrombinase nature of Der p 1 3 and that the thrombin substrate fibrinogen and its cleavage products are putative endogenous ligands of TLR4 21, 22 we were interested to investigate whether TLR4 transduced ROS generation by HDM allergens (i.e., Der p 1). Figure 8C shows that responses to mixed HDM allergens or purified Der p 1 (at a concentration matching that in the complete allergen mixture) were blocked in an identical manner by TAK‐242, a compound which prevents binding of the TIRAP and TRAM adaptor proteins to cys747 in TLR4 23. The similarity in the inhibition profiles reinforce the key role in TLR4 activation played by Der p 1 compared to other component HDM allergens that was suggested by the experiments in Figure 8A,B. We next examined the effect of TLR4 gene silencing on the response to mixed allergens (Fig. 8D). The response was inhibited, providing additional confidence that the mechanism was operating through TLR4. Silencing of prothrombin had a similar inhibitory effect (Fig. 8D).

**Figure 9:**
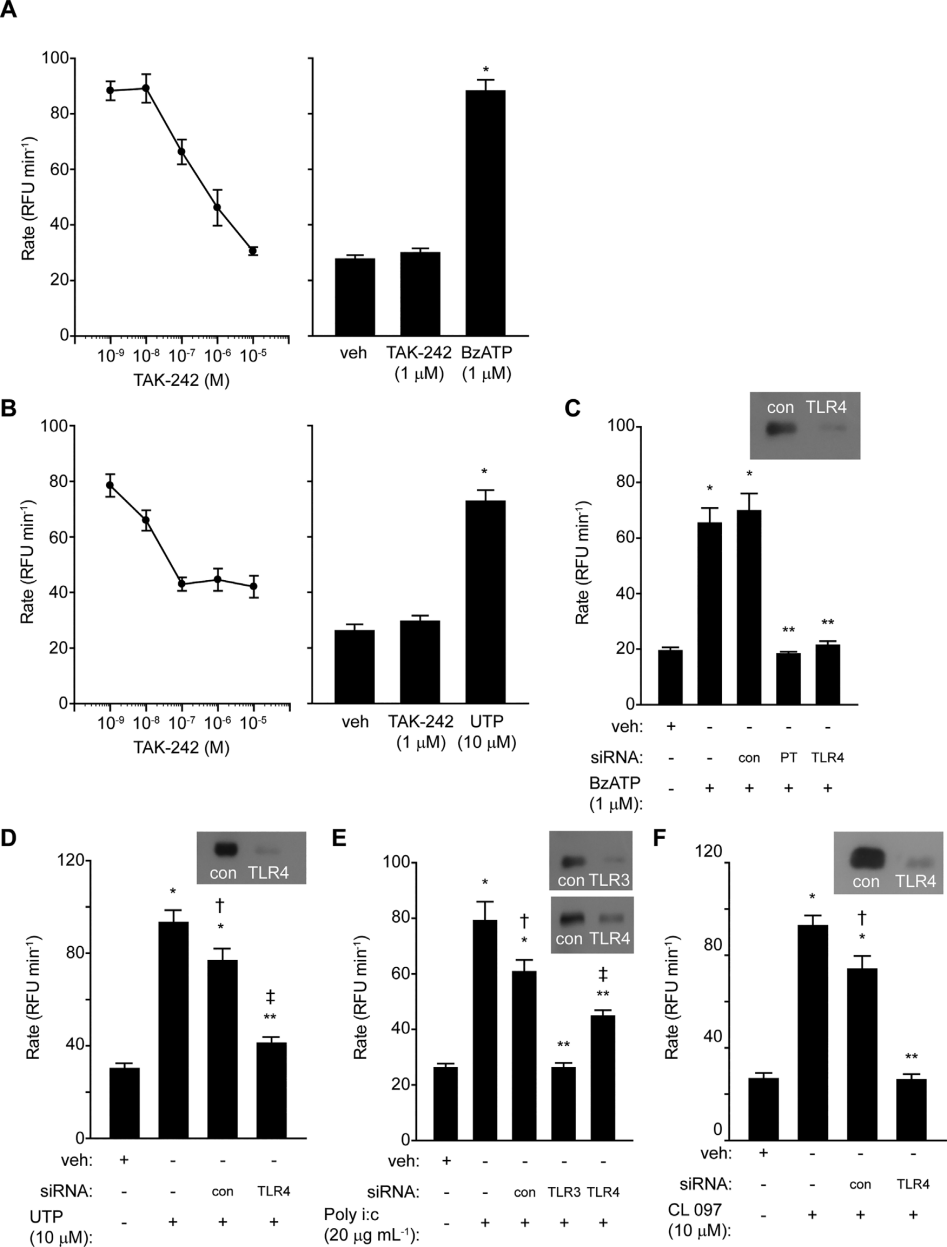
(A and B) TAK‐242 inhibits ROS generation by BzATP or UTP. The bar charts show the corresponding negative and positive controls (*P < 0.05 *v* vehicle (veh)). The effects of TAK‐242 were statistically significant at 10^−7^ M and higher. (C and D) Cell silencing of prothrombin (PT) or TLR4 attenuates responses to BzATP or UTP (*P < 0.05 *v* veh; **P < 0.05 *v* BzATP or UTP in non‐transfected cells or transfection controls, ^†^ *P* < 0.05 *v* response in non‐transfected cells, ^‡^ *P* < 0.05 *v* veh). (E and F) Cell silencing of TLR3 or TLR4 inhibits responses to poly i:c or CL 097 (*P < 0.05 *v* veh, **P < 0.05 *v* non‐transfected cells or transfection controls, ^†^ *P* < 0.05 *v* CL097 in non‐transfected cells or transfection controls, ^‡^ *P* < 0.05 *v* veh).

The finding that poly i:c and CL 097 operated intracellular ROS production in an argatroban‐sensitive and prothrombin‐dependent manner (Fig. 7) was surprising and prompted examination of whether ROS production through TLR3‐ or TLR7‐dependent routes also involved TLR4. We found that responses to poly i:c, CL 097 BzATP and UTP were reduced in cells in which TLR4 was inhibited by TAK‐242 or silenced by siRNA (Fig. 9A–F). This is consistent with a cyclical pathway which regenerates itself via thrombin formation as previously reported for HDM/Der p 1 3, 6. Silencing of TLR4 was less effective against poly i:c then for other stimuli (Fig. 9E), but the significance of this is unclear.

## Discussion

This study provides the first mechanistic linkage between a globally important inhalant allergen, the stimulation of viral RNA sensor TLRs, the activation of TLR4 and the intracellular generation of ROS in human airway epithelial cells. By combining physical presentation with non‐dispensable signaling events, the airway epithelium has a decisive role in networking the connection between innate and acquired immune responses to inhaled threats through communication with dendritic cells and, thereby, T‐lymphocytes 1, 17, 19, 20, 24, 25, 26. Our findings newly identify that the protease allergen Der p 1, recently revealed to be a prothrombinase 3, or signaling from either TLR3 or TLR7 eventuates in the activation of TLR4 through steps regulated by MYLK and ROCK and which require the gating of pannexons, purinoceptor signaling and endogenous thrombin formation. Pannexon opening is crucial to both the allergen and viral RNA sensor activation mechanisms, indicating that signaling convergence occurs upstream from this step.

**Figure 10:**
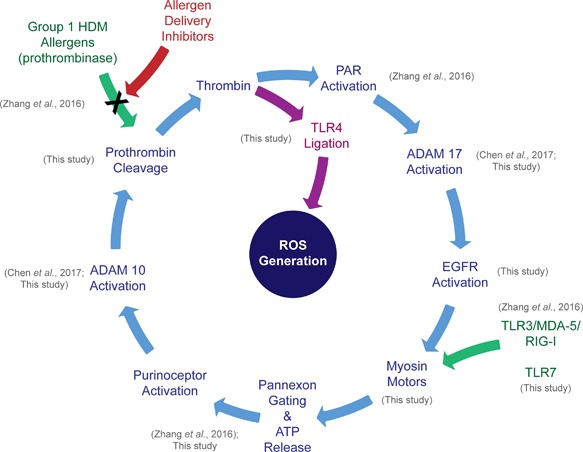
Schematic representation of ROS production initiated by Group 1 HDM protease allergens or viral RNA sensor ligation based on current understanding from data described in the present study and earlier work, as indicated. The activation of TLR4 occupies a central role in ROS generation initiated by signals of either origin. Kinetic studies will be necessary to confirm the reaction sequence and additional work investigating reaction participants in key steps (e.g., the ligation of EGFR and TLR4) are required. Other unresolved issues concern the subcellular provenance of ROS generation and cross‐talk between different mechanisms, the conditional operation of pannexons for ATP release and why signaling at this stage appears to involve both G‐protein‐coupled and ligand‐gated ion channel purinoceptors.

Our previous work showed that additional to the generation of ROS through TLR3, poly i:c also stimulated ROS formation through MDA‐5 and RIG‐I 3, suggesting that signaling through these transducers also involves pannexon gating and thus pathway convergence. Our present understanding of the pathways is summarized in Figure 10 which defines a context for the more detailed kinetic investigations that will be necessary to confirm mechanisms, fully understand the provenance of ROS being generated and further explore contributions to the establishment and exacerbation of allergic airways disease. The convergence of signaling pathways activated by an innate response to RNA viruses (which is protein synthesis and transport‐dependent 3), with the pathway arising from the thrombin‐dependent activation of PAR 1 and PAR 4 by Der p 1 (which is less dependent on protein synthesis and transport 3) is noteworthy for several reasons. Interactions between inhalant allergens and respiratory viruses are risks for the initiation of asthma exacerbations 8, 27, and viral replication induces epithelial remodeling and mucous metaplasia 28. It will therefore be of particular interest, although technically challenging, to investigate the operation of these events in humans with active disease. This study provides an embarkation point for those studies.

Within the constellation of HDM allergens the Group 1 cysteine proteases, exemplified by Der p 1, identify as the principal components possessing the bioactivity responsible for the generation of intracellular ROS 3. This arises from their prothrombinase nature which leads to PAR activation with the subsequent generation of ROS 3. Herein, we demonstrated that SGUL 1733, an ADI which targets Group 1 HDM allergens, blocked ROS generation by a natural mixture of *D. pteronyssinus* HDM allergens, as did pharmacological inhibition or gene silencing of TLR4. Whereas TLR4 is conventionally regarded as a receptor for exogenous ligands such as bacterial lipopolysaccharides (LPS), the blockade by SGUL 1733 shows that any role for canonical activation by LPS in this system is nugatory, and indeed our previous work has shown that the allergen‐dependent ROS generation occurs below the threshold for LPS responses 6. Activation of TLR4 may also occur through the formation of endogenous ligands, among which are the thrombin substrate fibrinogen and its cleavage products 21, 22, 29. Most fibrinogen is synthesized by the liver, but airway epithelial cells—from which it is secreted vectorially in a microtubule‐dependent manner—are an additional source of all three of its component chains 30. Notably, fibrinogen cleavage products and TLR4 activation account for the development of pathophysiological markers of allergic responses in mice exposed to proteases derived from *Aspergillus oryzae* 31. This encourages a parallel with HDM where a role for fibrinopeptides in intracellular ROS generation is suggested by various facets of our data, particularly the susceptibility of TLR3‐, TLR7‐, and purinoceptor‐evoked responses to the silencing of prothrombin expression or the direct inhibition of thrombin. However, ensemble participation of other endogenous TLR4 ligands cannot be excluded and further insight will require an examination of candidate ligands released from epithelial cells by thrombin or the “sheddase” activity of ADAM 10 which is an additional component of these responses 6.

In mice, the expression of TLR4 on epithelial cells is required for the development of allergic sensitization to HDM 12 and its ligation leads to an activation of cells by IL‐1α culminating in the release of GM‐CSF and IL‐33 13. Neutralization of any component of this cytokine triad or deletion of TLR4 thwarts the progression from an innate response to the development of allergic sensitisation 12, 13, but despite these insights it is not apparent how HDM allergens activate epithelial TLR4 to trigger this progression. Similarity between the HDM allergen Der p 2 and myeloid differentiation protein‐2 (MD‐2, lymphocyte antigen 96), a co‐receptor protein for LPS responses, has been assumed to implicate LPS and Der p 2 as key determinants of why TLR4 activation is essential for the development of allergy to HDM, and this is consistent with the ability of LPS to drive allergic immunity under certain conditions 32, 33. However, our data suggest an alternative explanation where the critical step is thrombin formation by the direct prothrombinase activity of Group 1 HDM allergens which then enables endogenous TLR4 ligand generation in the airway epithelium. In mice, inhibition of the proteolytic activity of Der p 1 prevents the development of sensitization to mixed HDM allergens or unrelated bystanders which rely on collateral priming for allergic responses 34, 35, 36, 37, a result which suggests that any involvement of Der p 2 and/or LPS in determining allergic sensitization is dispensable in the presence of protease allergens. Moreover, it is noteworthy that purified Der p 2 fails to emulate the production of intracellular ROS by mixed HDM allergens 3 which, as demonstrated herein, is a TLR4‐dependent response dominated by the proteolytic action of Der p 1.

Surprisingly, pharmacological and gene‐silencing experiments revealed that ROS generation by poly i:c and CL 097 also stimulated thrombin formation and resulted in sustained, TLR‐4 dependent intracellular ROS generation. The clear implication is that supplemental to an exogenous prothrombinase (i.e., Der p 1) which triggers ROS production, downstream signaling unleashes endogenous prothrombinase activity as a transductional mode which leads to TLR4 ligand generation. Intracellular ROS generation in airway epithelial cells by HDM allergen or viral RNA sensor activation thus share, through confluent signaling, pleiotropic operational elements (thrombin, ADAM 10, EGFR, TLR4) fundamental to the progression of allergic sensitization and asthma exacerbations.

ROS are compelling mediators of interactions between allergens and viruses through direct and indirect effects on gene expression 7 or the release of constitutively expressed regulators of type 2 innate immune responses 38, 39, 40. Of interest in the latter regard is IL‐33 which is implicated in asthma susceptibility by genome‐wide association studies and whose ROS‐ and EGFR‐dependent release is enhanced in asthma 9, 41, 42. Like IL‐4 and IL‐13 10, IL‐33 suppresses innate antiviral immunity 8 and is thus part of a signaling nexus which links key allergens with inflammatory signaling from viruses.

Our data highlight a significant role for pannexons in the confluent signaling response through which Der p 1 and viral RNA sensors generate intracellular ROS. In non‐excitable cells pannexons have a low open probability and, in contrast to excitable tissues, membrane potential is unlikely to control their gating. Various actions may lead to pannexon opening in non‐excitable cells but their significance, especially under physiological conditions, remains an area of uncertainty and thus active interest 43. These actions include proteolytic cleavage of the intracellular C‐terminus of pannexin‐1 by caspase 3 or caspase 7 which, because the C‐terminus of each pannexin‐1 protein normally occludes the lumen of the pannexon hexamer, results in a constitutively open channel that commits the cell to apoptosis 44, 45, 46.

Mechanical stimulation triggers calcium flux and opens pannexons as demonstrated in cells that have been artificially swollen and stretched 47, 48, 49. Thus, other types of stress‐related activation could be anticipated. In airway epithelial cells, Der p 1 evokes changes in the actin cytoskeleton which accompany the cleavage of tight junctions (TJs) and the concomitant non‐specific increase in epithelial permeability 19. This association initially suggested that the cytoskeletal changes arose because TJ cleavage initiated outside‐in cellular signaling which triggered apoptosis 18, 20. However, the cytoskeletal changes are widespread across an epithelial surface exposed to HDM allergens, whereas apoptosis occurs discretely 20. Moreover, apoptosis occurs in cells constitutively lacking TJs, or after their removal 20, indicating that the cytoskeletal rearrangements occur through other mechanisms with different consequences. This prompted us to consider mechanical stress arising from actomyosin contractility as a mode of pannexon opening in cells activated by HDM allergens or by ligation of virus‐sensing TLRs. While our findings show that enzymes which control the phosphorylation state of myosin light chains, and which thus affect the operation of myosin motors, have striking effects on ROS production by these stimuli, a limitation of our study is the absence of direct measures of myosin phosphorylation. Thus, further work with other approaches will be required to confirm our conclusions.

Our new data implicate both ADAM 10 and EGFR in ROS generation after TLR3/TLR7 ligation (Fig. 10). ADAM 10 is pertinent to the development of allergy through multiple mechanisms 50, 51, 52. Our previous work with Der p 1 suggested that ADAM 10 activation lies downstream from ATP release and amplifies the conversion of prothrombin to thrombin 6. The present studies demonstrate that this amplification also occurs after ligation of TLR3 or TLR7 and is thus a distinct endogenous cellular mechanism for thrombin formation in which ADAM 10 behaves directly or indirectly as a prothrombinase, mirroring snake venom proteases of family M12B to which it is related.

This concept of an essential endogenous prothrombinase‐dependent step (Fig. 10) provides a new understanding of our forerunner work which established that ROS production triggered by TLR3 ligation is blocked by PAR 1 antagonists 3. The new findings further support intracellular ROS production by Der p 1 or TLR3/TLR7 ligation being a cyclical chain reaction which features extensive receptor cross‐talk. This cyclical nature creates the potential for its regenerative operation through thrombin formation. Reasons for the dual involvement of thrombin‐sensitive PARs and the thrombin‐dependent activation of TLR4 are currently unclear, but a plausible hypothesis under investigation is that the PAR‐mediated responses may terminate quickly due to receptor internalisation, whereas TLR4 stimulation facilitates a prolonged, recycling activation that is characteristic of the cellular behavior observed. Key questions about this nexus remain unresolved, not least the mechanistic details of its operational control in which defining the key oxidants and their provenance will be key.

In summary, our findings indicate that the gating of pannexons and TLR4 ligation are key to innate oxidative stress responses to a proteolytic inhalant allergen of clinical significance. This provides an unexpected link to mechanisms operated by the ligation of receptors known to be activated by respiratory viruses whose inflammatory effects are associated, at least part, with the exacerbation of asthma. The linkage between these events and their expected consequences suggest that novel inhibitors of Group 1 HDM allergens, exemplified here by SGUL 1733, may have useful profiles as novel therapies for asthma and allied allergic conditions. Clearly, it will now be interesting to examine these pathways in the context of active viral infections.

## Authors' Contributions

CR and DG conceived the study area and wrote the first draft of the manuscript. JZ, JC, SCM, CPB, and CR designed experiments and analyzed data. Reagents were created and experiments were conducted by JC, JZ, SCM, and CPB. Funding for the research was obtained by CR. All authors approved the final version of the manuscript.

## Conflict of Interest

The authors declare that no conflicts of interest exist.
